# Development of chitosan lipid nanoparticles to alleviate the pharmacological activity of piperine in the management of cognitive deficit in diabetic rats

**DOI:** 10.1038/s41598-024-58601-x

**Published:** 2024-04-08

**Authors:** Asmaa Badawy Darwish, Amira Mohamed Mohsen, Shaimaa ElShebiney, Rania Elgohary, Mostafa Mohamed Younis

**Affiliations:** 1 Pharmaceutical Technology Department, National Research Centre (Affiliation ID: 60014618), El-Buhouth St., Dokki, Giza, 12622 Egypt; 2 Narcotics, Ergogenics, and Poisons Department, National Research Centre (Affiliation ID: 60014618), El-Buhouth St., Dokki, Giza, 12622 Egypt

**Keywords:** Piperine, Chitosan lipid nanoparticles, Diabetic cognitive deficit model, BDNF, Y-maze, Spatial memory, Biomarkers, Drug delivery, Pharmaceutics

## Abstract

The aim of the present study was to prepare and evaluate Piperine (PP) loaded chitosan lipid nanoparticles (PP-CLNPs) to evaluate its biological activity alone or in combination with the antidiabetic drug Metformin (MET) in the management of cognitive deficit in diabetic rats. Piperine was successfully loaded on CLNPs prepared using chitosan, stearic acid, Tween 80 and Tripolyphosphate (TPP) at different concentrations. The developed CLNPs exhibited high entrapment efficiency that ranged from 85.12 to 97.41%, a particle size in the range of 59.56–414 nm and a negatively charged zeta potential values (− 20.1 to − 43.9 mV). In vitro release study revealed enhanced PP release from CLNPs compared to that from free PP suspensions for up to 24 h. In vivo studies revealed that treatment with the optimized PP-CLNPs formulation (F2) exerted a cognitive enhancing effect and ameliorated the oxidative stress associated with diabetes. PP-CLNPs acted as an effective bio-enhancer which increased the potency of metformin in protecting brain tissue from diabetes-induced neuroinflammation and memory deterioration. These results suggested that CLNPs could be a promising drug delivery system for encapsulating PP and thus can be used as an adjuvant therapy in the management of high-risk diabetic cognitive impairment conditions.

## Introduction

Hyperglycaemia, or elevated blood sugar levels, is a common symptom of diabetes mellitus (DM), a metabolic disorder caused by deficiencies in insulin secretion, insulin action, or both. Approximately 90–95% of persons with diabetes have type 2 diabetes. Numerous consequences, such as dyslipidaemias, cardiovascular disease, a higher risk of peripheral neuropathy, and diabetic encephalopathy, which are linked to an increased incidence of cognitive issues, are typically present with chronic hyperglycaemia^1,2^. Impaired cognitive functions in diabetic patients are an enormous problem. Millions of people around the world suffer from dementia, early cognitive decline or depressive syndrome in relation to long periods of diabetes, even though they are receiving antidiabetic drugs. The metabolic condition resulting from type 2 diabetes is known to be associated with an increased risk of multitude of neurodegenerative disorders such as Alzheimer's disease, Huntington’s disease (HD) amyotrophic lateral sclerosis, and Parkinson’s disease^3,4^. It has been attributed to the harm resulting from high blood glucose level which in turn presents oxidative stress, and neuroinflammation, causing neuronal cell death and cognitive deterioration, particularly in specific areas such as hippocampus^5^. Management of diabetic encephalopathy or neuropathy necessitates the use of other therapeutic agents in addition to antidiabetic agents, thus increasing the body burden of chemical synthetic drugs^6^.

One of the main plant alkaloids, Piperine (N-acylpiperidine) (PP), is derived from long and black peppers (*P. longum *Linn and *P. nigrum* Linn)^7^. Previous reports have described various pharmacological activities of PP, including antiapoptotic^8^, anti-inflammatory^9^, antioxidant^10^, antidepressant^11^, antitumour^12^, and antihypertensive^13^ effects. In 1979, PP was scientifically established as a potent bioenhancer^14^. A bioenhancer is an agent that increases the bioavailability and bio efficacy of another combination medicine while exhibiting no normal pharmacological effect at the dose utilized^15^. It works by boosting fast absorption or suppressing metabolizing enzymes involved in medication biotransformation^15,16^. However, PP is a sparingly soluble lipid in water, and consequently, its poor oral bioavailability limits its pharmaceutical application^17^. This constraint can be solved with the use of drug delivery methods that increase the water solubility of the loaded medication and achieve sustained release patterns.

Although there has been much research on innovative drug delivery strategies, oral administration is still the most efficient, least complicated, and least likely to cause side effects^18,19^. Poor bioavailability is the primary drawback of oral administration, but the use of nanocarriers as oral medication delivery systems has gained recognition as a way to overcome these restrictions^20^. Nanoparticles have demonstrated superiority over traditional drug delivery systems because they can increase the solubility of hydrophobic medicines, have longer circulation times, and provide targeted drug administration^21^.

Many pharmacological researchers are becoming increasingly interested in the natural polymer chitosan^22,23^. Chitosan (CS) is a natural polysaccharide produced by partial deacetylation of chitin^24^. Chitosan is widely used in a variety of industries, including cosmetics, food, and agriculture, due to its outstanding biocompatibility, toxicological safety, and biodegradability^25,26^. Its cationic mucoadhesive properties maintain a regulated sustained release pattern of drugs^27^. It effectively delivers drugs to the gut membrane and enhances their bioavailability and permeability. Some of the CS nanocarriers have been stabilized by modifications^28^ or crosslinking^29^ to develop chitosan lipid nanoparticles (CLNPs), which are desirable as oral drug delivery carriers. According to previous reports, the positively charged chitosan in CLNPs can bind to cell membranes, minimize the transepithelial electrical resistance (TEER) of cell monolayers, and increase paracellular permeability, thereby overcoming the limitations of conventional oral drug delivery. It has been demonstrated that chitosan solutions, depending on the molecular weight and degree of acetylation of chitosan, can improve transcellular and paracellular permeability in a reversible, dose-dependent way^30^.

The objective of the current study is the development of PP-CLNPs to investigate its pharmacological activity in a complicated type 2 diabetes model either alone or in a combination with the known antidiabetic agent metformin. In order to reach a system with optimal characteristics, PP-CLNPs were prepared and characterized for drug encapsulation efficiency (EE %), vesicle size (VS) and zeta potential (ZP). The in-vitro drug release behaviour was investigated for the selected formulation with optimum characteristics. The in-vivo efficacy of the selected formulation, compared to free PP and metformin, was evaluated on complicated type 2 diabetes model in rats.

## Materials and methods

### Materials

Piperine (purity: 98%, code A13510) was purchased from Alfa Aesar (Gmbh & Co KG, Karlsrushe, Germany). Stearic acid and low-molecular-weight chitosan (100,000–300,000 Da) were obtained from Acros Organics Co., Belgium. Tween 80 was purchased from Loba Chemie Co., India. Tripolyphosphate (TPP) was purchased from Sigma-Aldrich Chemie GmbH, Japan. A dialysis tubing cellulose membrane was obtained from Sigma Co., USA, and a molecular weight cut-off of 12,000-14,000 was used. Streptozotocin (STZ) was purchased from Sigma Aldrich Chemical Co., USA. Sodium citrate, citric acid and hydrochloric acid of analytical grade were obtained from Fluka, Germany. Commercial ELISA (enzyme-linked immunosorbent assay) kits were used for the measurement of malondialdehyde (MDA), reduced glutathione (GSH), superoxide dismutase (SOD), brain-derived neurotrophic factor (BDNF) and the transcription factor CREB. All the other solvents were of analytical grade.

### Methods

#### Preparation of piperine-loaded chitosan lipid nanoparticles (CLNPs)

Oil-in-water homogenization was used to create PP-CLNPs, which were then crosslinked using an ionic crosslinking approach with some alterations^31,32^. Eight ml of 0.3% (v/v) acetic acid were used to dissolve the chitosan. Tween 80 was subsequently added to the chitosan solution while it was stirred magnetically at 1500 rpm to create a uniform aqueous solution. A few drops of a 1 M NaOH solution were added to the produced aqueous solution to bring its pH down to 5–5.2. After dissolving stearic acid and PP in one ml of methylene chloride, the organic solution was gradually added to the aqueous solution, and a high-speed homogenizer (Heidolph, Silent Crusher M, Germany) was used to run at 20,000 rpm for 5 min. The resulting emulsion was sonicated for 30 min at 40 °C in a bath sonicator (Elmasonic S 40H, Elma Schmidbauer GmbH, Germany) to disseminate the nanolipid particles within the shell of the polymeric medium and evaporate the organic solvent. To produce PP CLNPs, the mixture was sonicated and then gradually mixed with 2 ml of a tripolyphosphate (TPP) solution (pH 5, adjusted by 1 M HCl) and a chitosan cross-linker while being continuously stirred with a magnetic stirrer at 1500 rpm. In the absence of TPP, PP CLNPs were generated using the same method as CLNPs, except that 2 ml of distilled water was added following sonication in place of the TPP solution. The composition of the developed PP-CLNPs is displayed in Table 1.

**Table 1:** Composition and characterization parameters of the developed Piperine CLNPs.

Formulation	Stearic acid %(w/v)	Piperine % (w/v)	Chitosan % (w/v)	Tween % (w/v)	TPP % (w/v)	EE (%) ± SD	PS (nm) ± SD	PDI	ZP (mV) ± SD
F 1	0.05	0.05	0.2	0.2	0	96.23 ± 1.23	208.4 ± 47.08	0.488	− 43.9 ± 6.23
F 2	0.05	0.05	0.2	0.2	0.05	97.41 ± 0.55	293.2 ± 89.33	0.245	− 37.8 ± 7.76
F 3	0.05	0.05	0.2	0.2	0.1	96.69 ± 0.33	414 ± 93.63	0.798	− 22.9 ± 4.92
F 4	0.05	0.05	0.2	0.4	0	90.46 ± 2.69	119 ± 18.87	0.115	− 35.1 ± 7.75
F 5	0.05	0.05	0.2	0.4	0.05	95.80 ± 1.49	167.8 ± 43.20	0.374	− 31.9 ± 6.36
F 6	0.05	0.05	0.2	0.4	0.1	90.16 ± 2.15	332.6 ± 23.67	0.596	− 25.9 ± 12.2
F 7	0.05	0.05	0.2	0.6	0	85.12 ± 5.81	59.56 ± 9.94	0.172	− 25.6 ± 4.69
F 8	0.05	0.05	0.2	0.6	0.05	87.75 ± 2.57	114 ± 28.76	0.368	− 33.5 ± 2.38
F 9	0.05	0.05	0.2	0.6	0.1	86.35 ± 0.59	134 ± 31.35	0.639	− 20.1 ± 4.56

Data were displayed as mean ± SD (n = 3).

#### Characterization of piperine-loaded CLNPs

##### Determination of the entrapment efficiency percentage (EE%)

Cooling centrifugation at 7000 rpm and − 4 °C using a chilled centrifuge (Union 32R, Hanil Co., Korea) separated the produced PP-CLNPs from free PP for 30 min. The pellets were rinsed twice with 50 cm^3^ of distilled water before being centrifuged for 30 min. The washing procedure was used to verify that all free drugs were removed from the empty space between nanoparticles. The supernatants were collected, and the amount of unentrapped PP in the supernatant was determined using a UV‒Vis recording spectrophotometer (UV-2401 PC, Shimadzu Co., Japan) at 342 nm. The measurements were made in triplicate, and the percentage of drug entrapment efficiency (EE%) was computed as follows:

$$ EE\% = Total\;amount\;of\;PP\;added - Amount\;of\;unentrapped\;PP{/}Total\;amount\;of\;PP \times 100. $$

##### Determination of particle size (PS), polydispersity index (PDI) and zeta potential (ZP)

A zeta-sizer (Nano Series ZS90, Malvern Instruments Ltd., Worcestershire, UK) was used to measure the PS, PDI, and ZP of the PP-CLNPs via dynamic light scattering (DLS). With double distilled water, each mixture was suitably diluted (1:50). Every measurement was calculated using the mean of three separate, independent samples. The best formulations in terms of EE%, PS, PDI, and ZP were chosen for further research.

#### In vitro release profile

The dialysis bag diffusion technique was used to study the in vitro release characteristics of PP from different CLNP formulations as well as from free drug suspensions^33,34^. The selected PP-CLNP formulations were resuspended in distilled water. A pre-soaked cellulose dialysis bag was filled with a volume equal to 2 mg of PP from each sample, and the bag was sealed on both ends. After that, the bags were placed in tightly sealed plastic containers that contained 100 ml of the release medium, which was made of phosphate buffer (pH 7.4) with 10% (v/v) ethanol to maintain the condition of the PP sink. The containers were maintained using a thermostated shaking water bath (Memmert, SV 1422, Germany) at 37 ± 0.5 °C and 100 rpm of rotation. To maintain the sink condition, 2 ml of the release medium was collected at predefined intervals and replaced with the same volume of fresh media. A spectrophotometric test was used to analyse the samples at 342 nm. The ratio of the amount of released PP to the initial amount of PP in the dialysis bag was used to compute the cumulative percentage of PP released at each time interval. The mean of three values ± SD was used to represent the results.

To ascertain the mechanism of PP release from the chosen formulations, kinetic analysis was used for the in vitro PP release data. Several kinetic equations were used to fit the data: the zero-order model (% cumulative drug released vs. time), the first-order model (log% cumulative drug remaining vs. time), the Higuchi model (% cumulative drug released vs. square root of time), and the Korsmeyer–Peppas model, which included the percentage of cumulative drug released up to 60% (log% cumulative drug released vs. log time)^35^. The appropriate release model was identified based on the regression coefficient (R^2^) derived from linear regression analysis of the release data. The best fit model was determined to be the release model, with an R^2^ value near one. For additional characterization, the PP-CLNP formulation that showed the appropriate release pattern was selected.

#### Characterization of the selected piperine-loaded CLNPS

##### Transmission electron microscopy (TEM)

The morphological properties of the selected PP-CLNPs, such as their form and size, were evaluated via TEM. Before evaluation, the samples were suitably diluted with distilled water. One drop of the sample was adsorbed on a copper grid and dried for 10 min. The sample was then stained with 1% phosphotungstic acid and air-dried at room temperature for 10 min. The samples were next investigated using TEM (JEOL, JEM-1230, Tokyo, Japan) at a high voltage of 160 kV.

##### Fourier transform infrared (FT-IR) spectroscopy analysis

An FT-IR spectrophotometer (JASCO 6100, Tokyo, Japan) was used to analyse the PP-CLNPs, which were chosen to identify any potential chemical interactions between their constituent parts. This finding verified that the lipid nanoparticles had hybridized. To prepare the pellets, chitosan, TPP, stearic acid, PP, and freeze-dried PP-loaded CLNPs were combined individually with KBr and compacted in a hydraulic press for 2 min at a pressure of 200 kg/cm^2^. Every sample KBr pellet was scanned at a wavenumber range of 4000–400 cm^−1^ on a blank KBr pellet background.

##### X-ray powder diffraction

XRPD was used to assess samples of PP, stearic acid, chitosan, TPP, selected PP-CLNPs, and physical combinations (approximately 200–300 mg each). An X-ray powder diffractometer (PANalytical EMPYREAN, UK) was used to produce diffraction patterns. The X-ray generator used copper Ka lines as the radiation source and ran at 45 kV tube voltages and a 30-mA tube current. In step scan mode, the diffraction angle ranged from 4 to 80° for (2), with a step size of 0.026° (2) and a step time of 21.42 s.

#### In vivo study

##### Animals

Male Wistar albino rats weighing 210–230 g (6–7 months old) were obtained from the National Research Centre's (NRC) animal house colony (Dokki, Giza, Egypt). Standard environmental conditions were maintained throughout the study, including controlled ambient temperature (25 ± 2 °C), relative humidity (35–50%) and unlimited access to water. A rodent chow diet with a high protein content (22%) and water were provided ad libitum. The investigation was carried out in compliance with the ethical criteria of the National research centre Animal Care Committee (NRC-ACUC), and the sample was granted clearance (clearance # 13144052022) prior to performance of the study. The experiments were consistent with the civil regulations of “Animal Welfare and the Institutional Animal Ethical Committee (IAEC)”, and were congruent with the “Animal Research: Reporting of In Vivo Experiments (ARRIVE)” guidelines.

##### Diabetes-induced cognitive deficit model

Diabetes was induced by an intraperitoneal injection of STZ (60 mg/kg) dissolved in 0.1 M sodium citrate buffer (pH 4.5)^36^. All animals were fasted overnight before STZ injection, and 48 h later, blood glucose was tested using a bio diagnostic kit. Only rats with blood glucose levels greater than 200 mg/dl were classified as diabetic and participated in the study. The rats were kept under observation for 14 days, after which the assigned treatment was started and continued for another 21 days.

##### Experimental design

Fifty-six rats were divided into 7 groups at random (n = 8). Control rats were injected with saline (4 ml/kg), diabetic rats were injected with STZ, diabetic rats were treated with free PP (4 mg/kg, p.o.), diabetic rats were treated with PP-CLNPs (4 mg/kg, p.o.)^37,38^, diabetic rats were treated with metformin (MET, 150 mg/kg, p.o.), diabetic rats were treated with MET + PP, and diabetic rats were treated with MET + PP-CLNPs. Rats were weighed at the start of the experiment and every week until the end of the study, and only initial and final weights were reported. The doses were given daily for 21 days and were adjusted to body weights weekly.

##### Behavioural analysis

Animals were subjected to behavioural evaluation at days 17, 18, 19, and 20. Tests were conducted on separate days to avoid animal tiredness and stress. For each group, treatment was given 1 h before the behaviour test session. To eliminate possible circadian effects on test outcomes, all tests were evaluated between 9:00 a.m. and 12:00 p.m.

**(a) Open field**

On day 17, the test began with the rat being placed in the centre of an open field arena (square wooden box 100 cm × 100 cm × 40 cm, divided into 16 squares). Over 5 min, the number of crossed squares and the vertical rearing activity were recorded by a blind observer.

**(b) Elevated plus maze**

On day 18, the animal was placed in the centre of the maze facing a closed arm. The number of entries into and time spent on each arm were scored for a 5 min test session*.*

**(c) Forced swimming test (FST)**

The FST has previously been used in rodents to measure immobility time (IT) to identify depressive-like behaviour. Individual rats were placed, on day 19, in a clear plastic cylinder (47 cm in height; 38 cm in diameter) with 38 cm of water (251 °C) for 6 min. The overall duration of immobility, including passive swimming, as well as the delay time, was calculated.

**(d) Spatial cognitive function through Y-maze**

The Y-maze is a simple two-trial recognition test used to assess spatial recognition memory based on rats' natural ability to explore new habitats^39^. The Y maze was divided into three arms at random: the new arm, the beginning arm, and the opposite arm. The Y maze experiment consisted of two steps. The new arm was closed in the first phase, and the rats were allowed to freely explore the other two arms for 10 min. Second, all of the arms were opened, and the rats were allowed to freely move between the three arms for 5 min. This session's first arm entry was observed. For each minute, the proportion of time spent in each arm and the percentage of entries made into each arm were recorded. The percentage of alternation was calculated.

##### Biochemical measurements

Fasting blood glucose levels were measured weekly in blood samples collected from the lateral tail vein via micro puncture according to the glucose oxidase principle using a fine test glucometer with a maximum reading capacity of 600 mg/dl (China) and reported as mg/dl. After the end of the experiments, blood samples were collected from the retro-orbital plexus under light anaesthesia. Sera were separated. Lipid peroxide levels were determined calorimetrically in serum as malondialdehyde (MDA) concentrations at 532 nm and are expressed as nmole MDA/ml^40^. The reduced glutathione (GSH) serum concentration was measured according to the methods of Bulaj et al.^41^. The GSH concentration was expressed as mmole/ml. The activity of superoxide dismutase (SOD) in the serum was determined using a colorimetric kit (Bio diagnostic, Egypt). The difference in absorbance after 5 min was recorded, and the activity was calculated and expressed as U/ml.

##### Determination of brain-derived neurotrophic factor (BDNF) and the transcription factor CREB

At the end of the experiments, the rats were euthanized under light anaesthesia, and the brains were extracted. The hippocampi were dissected and snap frozen at − 80 °C until ELISA analysis. ELISA kits for BDNF and CREB were used for hippocampal measurements (Elabscience, China).

#### Statistical analysis

The values are presented as the means ± standard errors. One-way analysis of variance (ANOVA) was used to compare means, followed by the Tukey–Kramer multiple comparisons test. For all the statistical tests, p ≤ 0.05 was considered to indicate statistical significance. All the statistical analyses were performed using GraphPad Prism software, version 8.

## Results and discussion

### Preparation of piperine-loaded chitosan-based lipid nanoparticles

Chitosan-based nanoparticles were developed by first homogenizing oil in water and then employing an ionic crosslinking approach^31^. Following their dissolution in a water-impermeable organic solvent, PP and stearic acid were emulsified with a chitosan solution in an aqueous medium. When the solvent was removed from the emulsion, stearic acid and PP precipitated together as one unit in the aqueous phase, creating a dispersion of nanoparticles^42^. The pH of the aqueous medium must be adjusted to properly prepare the generated lipid nanoparticles. The pKa values of stearic acid and chitosan are 4.75 and 6.5, respectively. Consequently, the carboxylic group of stearic acid is negatively charged, and the amino groups of chitosan are protonated at pH 5–5.2. This makes it possible for the amino groups of chitosan and the carboxylic groups of stearic acid to interact electrostatically, effectively coating lipid nanoparticles with chitosan. The polyanionic cross-linker TPP was used for CLNPs because it is biocompatible and nontoxic and exhibits effective electrostatic interactions at high enough concentrations. The positively charged amino groups in chitosan and the negatively charged TPP counterions mediate this connection^42^. Nine formulations (F1–F9) were developed at different ratios, and the most suitable formulations were filtered out.

### Characterization of the piperine CLNPs

#### Entrapment efficiency percentage (EE%)

Table 1 shows the EE% of all the developed formulations. High EE values ranging from 85.12 ± 5.81 to 97.41 ± 0.55% were revealed, which indicated good encapsulation of PP in the CLNPs. This could be explained by the hydrophobic nature of PP, as it has been previously reported that the lipophilicity of drugs plays a key role in determining the EE content^43^. Lipophilic drugs can migrate less to the aqueous phase after homogenization and sonication and have a stronger affinity for lipid nanoparticles^44^.

The influence of the TPP concentration on the drug EE% was investigated, and it was observed that increasing the TPP concentration to a particular ratio increased the nanoparticle EE%. This difference could be attributable to the decrease in size, which results in an increase in the surface area of the nanoparticulate matrix, allowing additional space for drug encapsulation. Because of the competing interaction between TPP and PP for chitosan binding, as we increased the TPP ratio (approximately 0.1%), the EE% decreased. Consequently, an optimal TPP ratio of 0.05% resulted in the development of homogeneously shaped and sized nanoparticles with the highest entrapment efficiency^45^.

#### Particle size analysis

Particle sizes for nanoparticle delivery can range from 10 to 1000 nm. The smaller particle has a bigger surface area and will be easier for the cell to absorb. As a result, faster release will be associated with smaller particles^46^. The investigation of the size of the PP-CLNPs revealed that the PS sizes ranged between 59.56 ± 9.94 and 414 ± 93.63 nm (Table 1). Generally, two factors affect the particle size of the prepared formulations: the ratio of Tween 80 to TPP. The results revealed that by increasing the tween ratio, the particle size decreased. Additionally, the results showed that the formulations (F7, F8 and F9) with the highest tween ratio (0.6% w/v) exhibited the smallest vesicle size. However, the formulations F1, F2 and F3, which had the lowest Tween 80 ratio (0.2% w/v), exhibited the largest vesicle size among the investigated formulations. These findings suggested that tween 80 may function as a stabilizing agent during the production of nanoparticles and reduce surface energy, which prevents crystal development^47^. This is consistent with the findings of prior studies, which demonstrated the capacity of surfactants to generate a thick protective layer around particles, stopping aggregation^34,48^.

Regarding the effect of the TPP ratio, the PS content of the developed nanoparticles increased with increasing TPP ratio within formulations with a constant tween ratio. This could be explained by the fact that as the TPP concentration increases, the particle size increases due to saturation of the interaction site on chitosan, resulting in the adhesion of surplus TPP anions on the positively charged surfaces of chitosan, which results in aggregation and increased particle size^49^. Furthermore, it was reported that smaller chitosan/TPP ratios make smaller nanoparticles, but only up to a point. If the ratio gets too small, the nanoparticles clump together or grow bigger instead^50^. This effect of the chitosan/TPP ratio on nanoparticle size has been confirmed in other studies^51,52^.

Furthermore, the results showed small polydispersity index (PDI) values (PDI < 0.5) for formulations prepared without TPP (F1, F4 and F7) and those prepared with a low TPP ratio (0.05% w/v) (F2, F5 and F8). It was reported that a PDI < 0.5 indicates homogeneity and a narrow distribution of the dispersed particles^33,53,54^. However, formulations that contained a high ratio of TPP (0.1% w/v) (F3, F6 and F9) exhibited high PDI values (PDI > 0.5), which indicated the formation of polydisperse particles.

#### Zeta potential

ZP is regarded as a crucial marker for the stability of dispersions. The size of the ZP represents the strength of the electrostatic attraction between dispersed particles with similar charges. When the ZP is low (≤ |20| mV), repulsion forces can be overcome by attractive forces, leading to aggregation. On the other hand, dispersion will promote stability and prevent aggregation at high ZP (≥ |20| mV)^55^. Table 1 reveals that all the PP-CLNPs had a negative charge ranging from − 20.1 ± 4.56 to − 43.9 ± 6.23. The produced formulations have good stability due to repulsion between positively charged nanoparticles, which prevents aggregation^56,57^.

It was also shown that increasing the TPP concentration decreased the ZP, which might be attributed to the presence of fewer unbound positively charged chitosan sites, as both TPP and PP compete for binding to free positive amino sites on chitosan^45^. The results revealed that F2, F5 and F8 had optimum EE%, PS and ZP values; thus, they were chosen as optimum formulations for further investigations.

### In vitro release studies of the selected formulations

**Figure 1:**
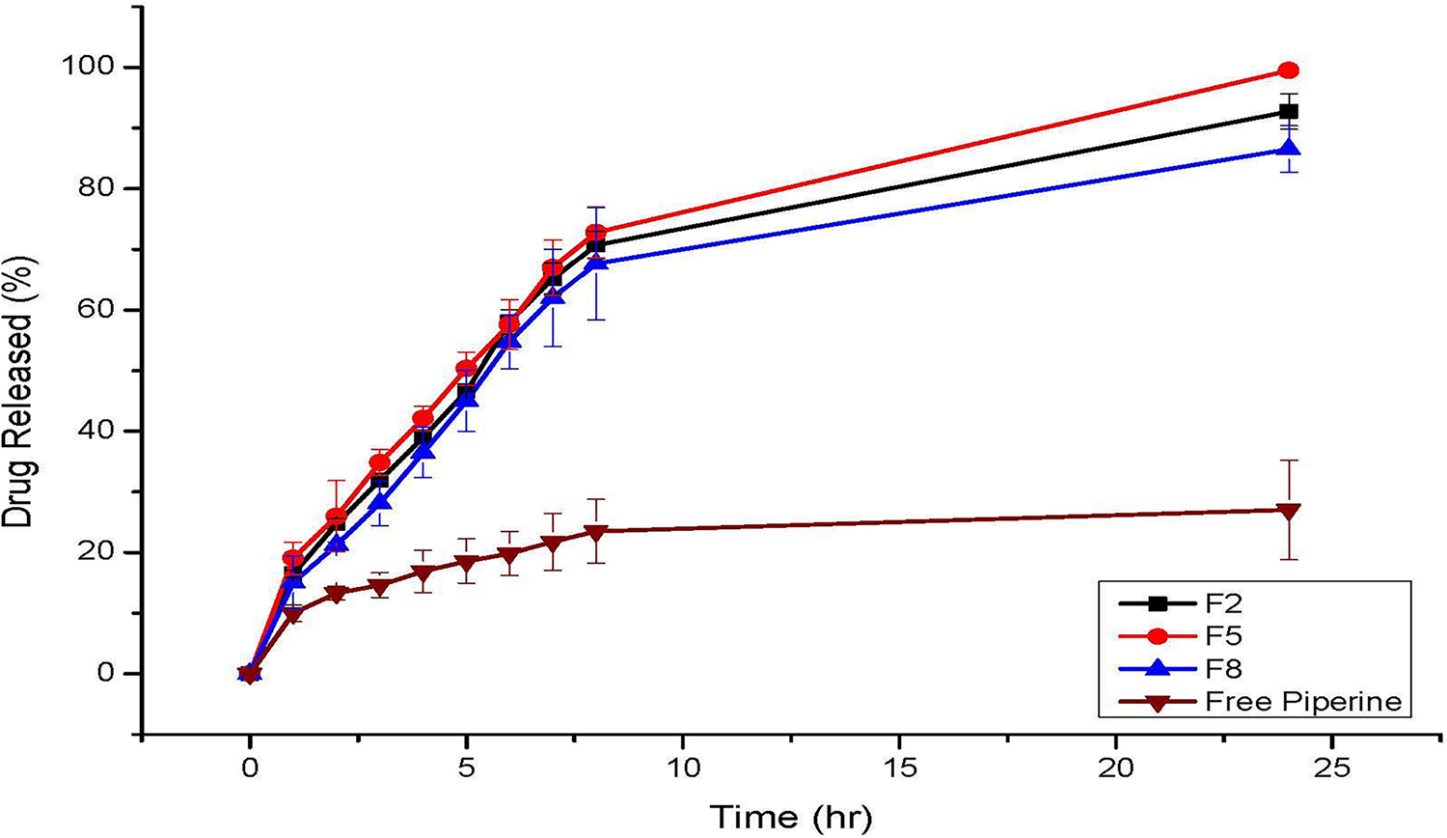
Release profiles of PP from the free drug solution and selected formulations of chitosan lipid nanoparticles (F2, F5 and F8).

Figure 1 shows the release profile of PP from the free drug solution and selected PP-CLNP formulations (F2, F5 and F8). The findings indicated that the chosen formulations exhibit improved PP release compared with that of the free drug. The findings demonstrated that after 24 h, 27.01 ± 8.91% of the free PP was released from the suspension. This value is significantly lower (p ≤ 0.05) than the cumulative percentage of PP released from the formulations investigated (86–99%). The reduced crystallinity of PP and the large surface area of the nanosized lipid nanoparticles. The reduced crystallinity of PP and the large surface area of the nanosized lipid nanoparticles could explain the significant increase in PP release from the chitosan lipid nanoparticle formulations. This finding is consistent with prior research on increasing the solubility of hydrophobic medicines by loading them into nanosized particles^42,58–60^.

All the investigated formulations exhibited two-stage drug release, an initial burst that lasted for the first 6 h, followed by slow and sustained release. These results are in accordance with earlier findings suggesting that the initial burst release of drugs can be attributed to the drug adsorbed on the surface of the nanoparticles, while the sustained release is due to the slow diffusion of the encapsulated drugs from the nanoparticle core^61,62^. Increasing the content of tween 80 decreased the release of PP from the nanoparticles. The inclusion of tween 80 reduced the particle size and increased the surface area coated with chitosan, which caused a decrease in drug release^63^.

Table 2 displays the results of a linear regression study of the mathematical models used to analyse the PP release data from the improved formulations F2, F5, and F8. Compared to those of the zero-order, first-order, and Higuchi kinetic models, the correlation coefficient (R2) values of F2, F5, and F8 revealed better fits to the Peppas model (R2 = 0.9469, 0.9533, and 0.9331 for F2, F5, and F8, respectively). Peppas theory states that drug release follows the Fickian diffusion process if n = 0.43, anomalous (non-Fickian) diffusion if n < 0.85, case II transport if n = 0.85, and super case II transport if n > 0.85^64,65^. The release exponent “n” values for F2, F5, and F8 were 0.0827, 0.0934, and 0.1072, respectively, revealing a non-Fickian diffusion-controlled release mechanism. F2 exhibited an optimum release profile and was chosen for further characterization and in vivo study.

**Table 2:** The calculated correlation coefficients and kinetics parameters of Piperine release profiles from the developed chitosan lipid nanoparticles.

Code	Zero order	First order	Higuchi	Peppas	
	R^2^			R^2^	n
F2	0.6758	0.5033	0.8315	0.9469	0.0827
F5	0.6161	0.4424	0.7846	0.9533	0.0934
F8	0.6879	0.5173	0.8377	0.9331	0.1072

### Transmission electron microscopy

**Figure 2:**
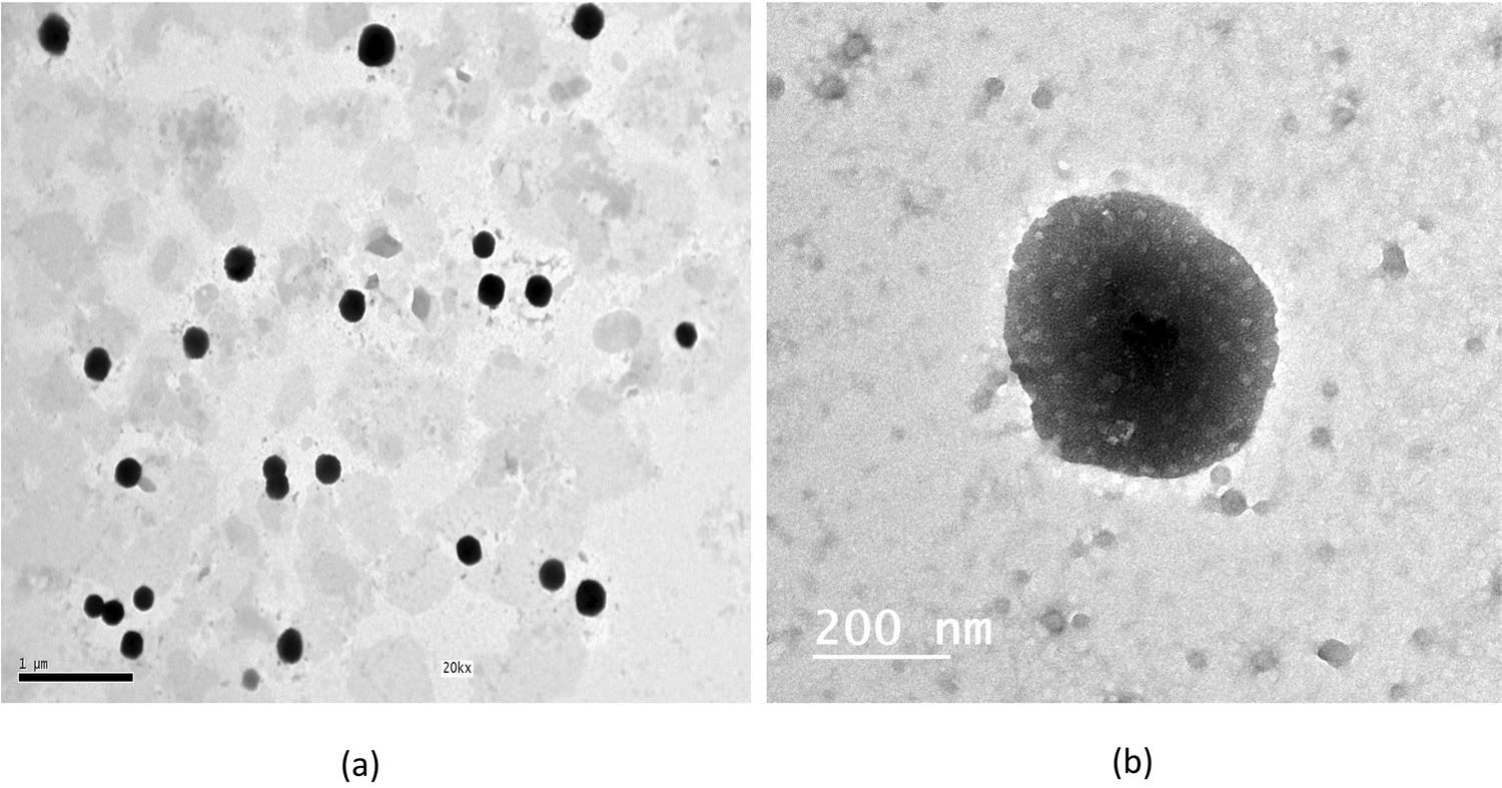
TEM micrographs of the selected chitosan lipid nanoparticles formulation F2.

The morphology of the selected PP-CLNPs formulation (F2) is depicted in Fig. 2. As shown in the micrographs, the nanoparticles were homogenously dispersed. The cells were spherical with smooth surfaces and were revealed as black dots^66^.

### Fourier transform infrared (FT-IR) spectroscopy analysis

**Figure 3:**
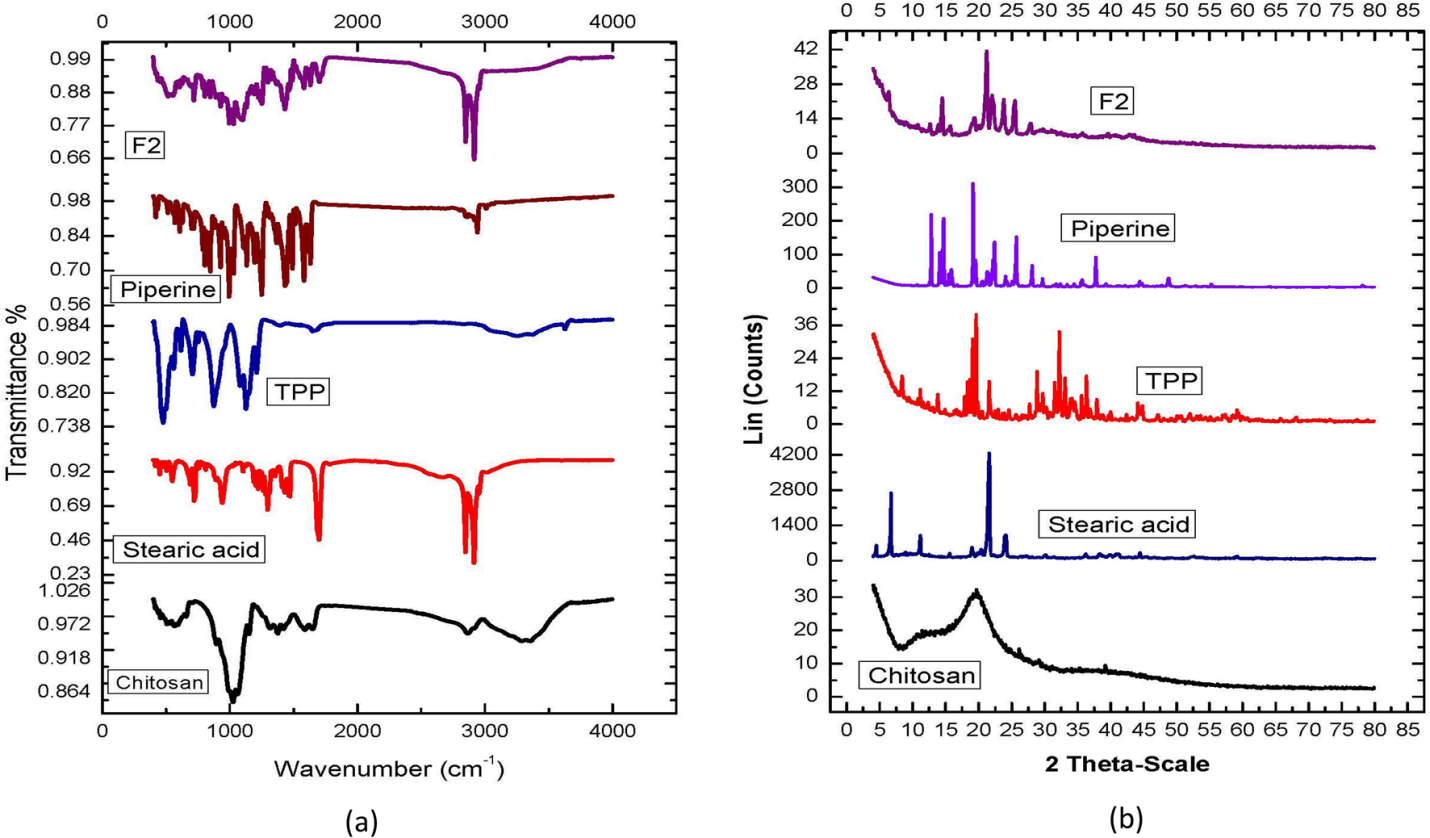
Characterization of different CLNPs components and optimized formulation through: (**a**) IR spectrum, (**b**) X-ray diffractometer.

The primary peaks of a material's distinct functional groups can be identified using Fourier transform infrared (FT-IR) spectroscopy, and their variations can be monitored within a designated fingerprinting area^67^. FT-IR was performed to investigate the interaction between PP and the various CLNP components. The FT-IR spectra of chitosan, stearic acid, TPP, free PP and the selected PP-CLNP formulation (F2) are shown in Fig. 3a. At wavenumbers between 4000 and 650 cm^−1^, the corresponding spectra were obtained^68^.

The FT-IR spectrum of chitosan revealed that hydroxyl and amine groups were represented by a broad band at 3353 cm^−1^, while –OH stretching was responsible for the peak at 2867 cm^−1^; the bending vibrations of N–H (N-acetylated residues, amide II band) at 1591 cm^−1^; and the absorption band of the carbonyl group (C=O) stretching of the secondary amide (amide I band)^69^ at 1625 cm^−1^. The N–H stretching of amide and ether bonds and N–H stretching (amide II band) are responsible for the peaks at 1450 and 1382 cm^−1^, respectively. The secondary hydroxyl group (typical peak of CHOH in cyclic alcohols, C–O stretch) and primary OH (typical peak of –CH2–OH in primary alcohol, C–O stretch) were the two peaks observed at 1060 and 1022, respectively^70,71^.

The FT-IR spectrum of stearic acid shows aliphatic C–H stretching, as revealed by the typical peak at 2847.24–2954.26 cm^−1^, whereas the carboxylic group's C=O stretching is represented by the strong peak at 1698.37 cm^−1^. For methylene groups (CH2), the peak at 1410.3–1468.81 cm^−1^ corresponds to C–H bending and scissoring^55^. The FT-IR spectra of TPP showed peaks at 1123.05 and 1211.82 cm^−1^, corresponding to the PO2 and P=O groups, respectively; the P-O group is represented by the peak^72^ at 909.81 cm^−1^.

The FTIR spectrum of PP showed distinctive peaks at 2925 cm^−1^, which are indicative of aromatic C–H stretching. C=O stretching is responsible for a significant absorption band in the vicinity of 1700 cm^−1^; the existence of this band suggests that a ketonic group (C=O) might exist within the compound. The presence of a ketonic (C=O) group was shown by the absorption at 1699 cm^−1^. The strong band at 1230 cm^−1^ clearly represents an ether group (methylene ether, –O–CH2–O–)^73^.

Some of the characteristic peaks related to chitosan disappeared or became weaker in the FT-IR spectra of the chosen PP-CLNP formulations. This could be because of multiple interactions (hydrogen bonding and electrostatic interactions) between chitosan and other components. The PP-loaded chitosan nanoparticles displayed some distinctive PP absorption bands, indicating that the PP molecule was confined to the polymeric network. However, other bands vanished because of the medication and polymers forming hydrogen bonds. Therefore, the production of PP-CLNPs was confirmed by FT-IR spectrum analysis^42,63^.

### X-ray diffraction analysis

The physical state of the PP-CLNP formulation (F2) and all the other components were assessed by investigating their crystallinity via XRPD analysis. As depicted in Fig. 3b, chitosan has a distinctive fingerprint of semicrystalline structures with a characteristic peak at 2θ = 19.94°^32,74,75^. TPP showed peaks at 2θ values of 11.2° and 20.06°, while stearic acid showed sharper peaks at 2θ values of 6.67° and 21.59°. The X-ray diffraction pattern of PP showed characteristic peaks at 2θ ranging from 7° to 23°, which is an indication of its crystallinity^76^.

The majority of the characteristic peaks for stearic acid, TPP, and chitosan are absent from the chosen PP-CLNPs formulation, as shown by an X-ray diffractometer, indicating a transition from a crystalline to an amorphous form. Furthermore, the characteristic peaks for PP were absent, suggesting that PP is trapped in an amorphous form within the lipid core^42,77^.

### In vivo study

Type 2 diabetes is linked to a number of structural and functional changes underlying impaired cognitive function. These abnormalities include alterations in neurochemical and signalling pathways, increased oxidative stress, decreased antioxidant activity, and mitochondrial dysfunction, which accelerate the aging of the central nervous system. The buildup of abnormal proteins, decreased DNA repair enzymes, and mild to moderate astrocyte and microglial activation are further causes of these abnormalities^78,79^. In allopathic medicine, metformin is a first-line treatment for the majority of people with type 2 diabetes^80^. However, most drugs target glycaemic control without paying attention to the accompanying complications. Nonetheless, these drugs were not shown to be completely effective; they induce unpleasant side effects, including gastrointestinal discomfort, hypoglycaemia, and lactic acidosis. Herbal medicines with few side effects have attracted much interest as adjuvants for the treatment of type 2 diabetes^81^. Additionally, Phyto molecules are capable of increasing the bioavailability and bio efficacy of a certain medicine when they are used in combination but exhibit no pharmacological activity at the dose utilized^82,83^. These adjuvants can be vital for the management of complications, especially cognitive-related disturbances. The current investigation demonstrated the possibility of increasing the therapeutic value of metformin in improving the prognosis of diabetic complications through combination with the developed PP CLNPs.

#### Effect of piperine on blood glucose level and body weight

**Figure 4:**
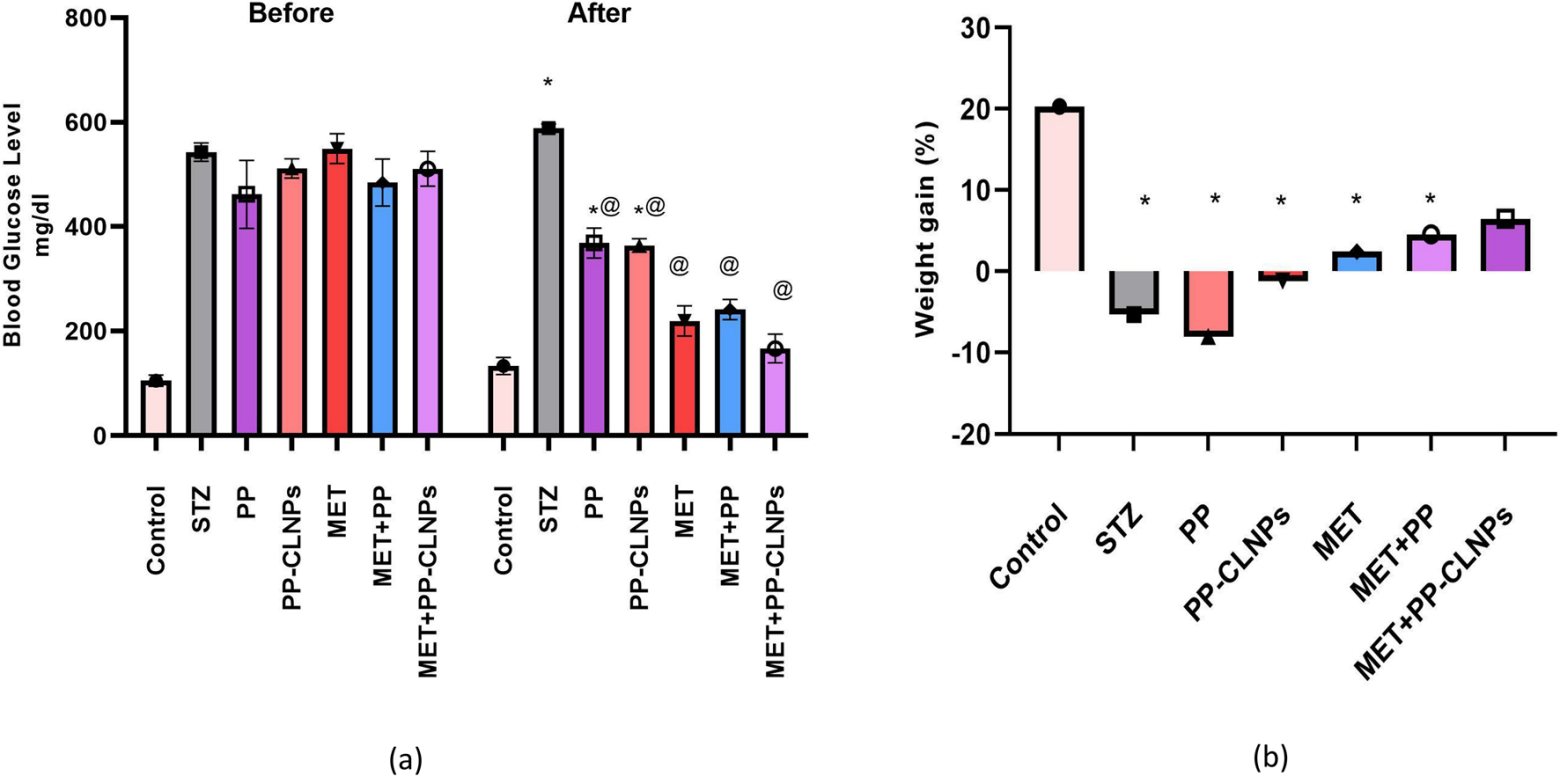
Effect of PP administration on (**a**) blood glucose level in diabetic rats, (**b**) body weight in diabetic rats. Data represented as mean ± SEM. *Denotes significance from control and # for significance from STZ at p < 0.05. One-way ANOVA followed by Tukey post hoc test was used for analysis.

STZ-induced diabetes resulted in a significant increase in the BGL accompanied by a significant decrease in body weight (p < 0.05). As shown in Fig. 4a, compared with no treatment (STZ), the administration of free PP or PP-CLNPs (F2) markedly reduced the BGL (⁓ − 35%, p < 0.05); however, the BGL did not decrease by less than 250 mg/dl, while MET alone or in combination with the PP combination enhanced the BGL (⁓ − 70%, p < 0.05), as the BGL was maintained at less than 250 mg/dl. This observation confirms the ineffectiveness of PP alone at the applied dose in the management of diabetes. The effect of combination was reflected on body weight represented in Fig. 4b, where MET + PP-CLNPs showed enhanced weight gain (4%) rather than PP alone (− 4%, p < 0.05) or metformin alone (− 1%, p < 0.05) and was not different from control level (p > 0.05). There was also a significant difference (P < 0.05) between the PP-CLNPs and the free PP. These results indicate that the PP-CLNPs significantly enhanced the activity of metformin in the reduction of BGL and enhancement of body weight^83^.

#### Effects of piperine on oxidative stress biomarkers

**Figure 5:**
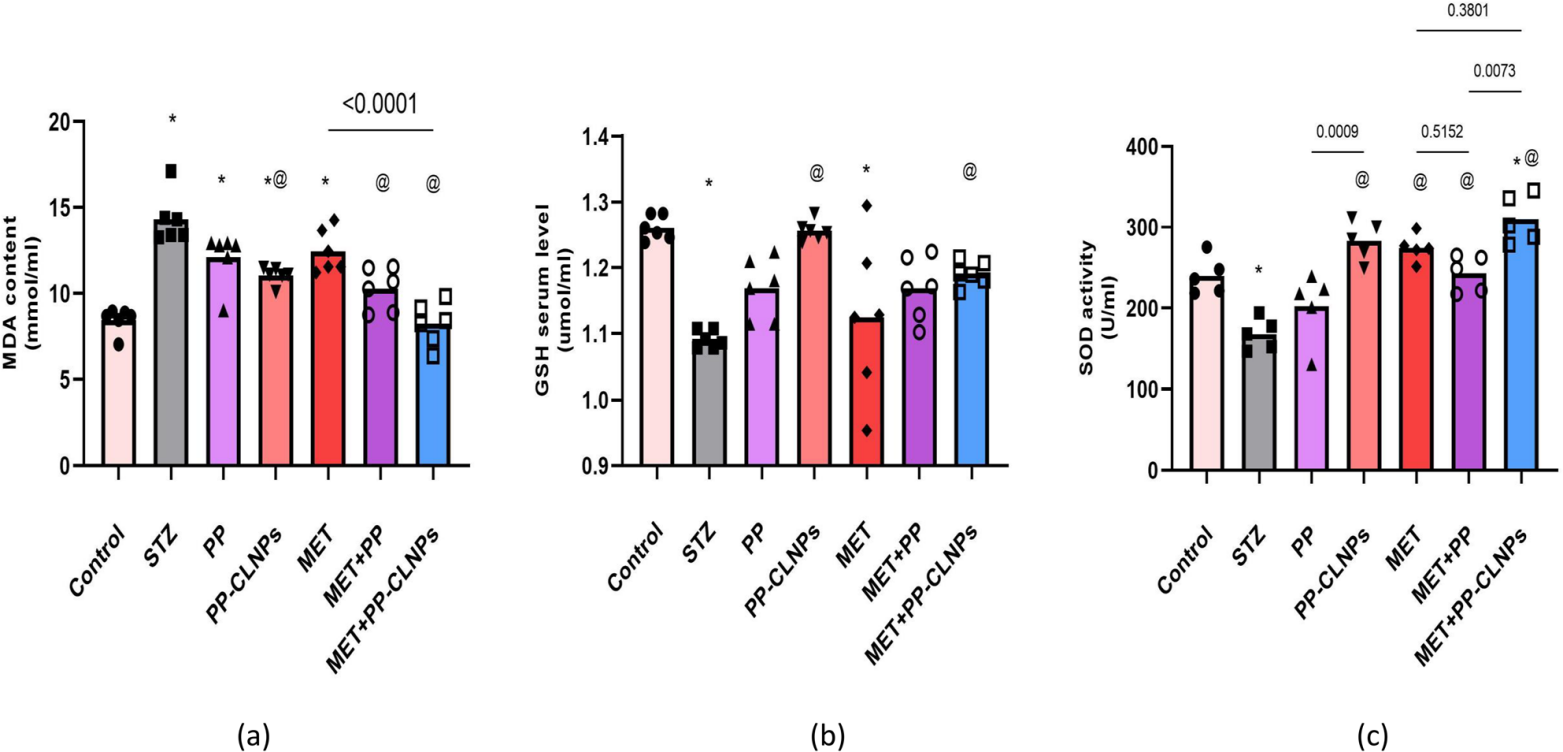
Effect of PP administration on the oxidative stress biomarkers in serum of diabetic rats. Data represented as mean ± SEM. *Denotes significance from control and # for significance from STZ at p < 0.05. One-way ANOVA followed by Tukey post hoc test was used for analysis.

Compared to those in the normal control group, STZ-induced diabetes induced the serum MDA concentration by about 70% (p < 0.05, Fig. 5). The MDA level is increased as an indication of lipid peroxidation after diabetic like condition induction. Treatment with free PP, PP-CLNPs or MET mildly reduced the serum MDA concentration in diabetic rats but did not normalize it (− 14%, − 21%, − 13%, p > 0.05 vs. STZ). Combining MET with free PP or PP-CLNPs significantly decreased the MDA concentration compared with that in the STZ and MET groups (− 29%, − 43%, p < 0.05) and was comparable to the control level (p > 0.05, Fig. 5).

The antioxidant molecule GSH was depleted in the serum in response to diabetes induction (− 14%, p < 0.05). Metformin did not increase the serum concentration (+ 4%, p > 0.05), but treatment with PP-CLNPs alone or in combination with MET boosted the serum GSH concentration compared with that in the STZ group (+ 15%, + 9%, p < 0.05).

Diabetes caused − 30% reduction in SOD activity (p < 0.05). Administration of free PP did not reverse the inhibition of enzymatic activity (+ 21%), but PP-CLNPs significantly elevated the enzymatic activity by 69% (p < 0.05). Combining MET with free PP or PP-CLNPs resulted in 45% and 85% increment of SOD activity (p < 0.05). The PP-CLNPs produced a more profound effect than the PP when administered alone or in combination with metformin (p < 0.05).

The superior effect of combination treatment with MET and PP-CLNPs could be attributed to the effect of incorporating PP into nanoparticles on enhancing their biological activity because it can improve their capacity to engage with a wide range of molecular targets, such as receptors, signalling molecules, kinases, transcription factors, cell cycle proteins, and inflammatory cytokines^84^. This could be due to the enhanced solubility of PP upon loading into lipid nanoparticles. It has been established that chitosan aids in intestinal absorption since positively charged chitosan interacts with tight junction proteins via transitory disruption, increasing cellular permeability^85^. Furthermore, the negatively charged sialic acid in mucus and the positively charged chitosan interact electrostatically to lengthen the GI tract's residence period, which in turn promotes the uptake of carriers in intestinal cells^86^. Many reports have shown that the activity of PP, an anti-inflammatory agent, can normalize oxidative stress biomarkers^87,88^. Various studies have shown that PP enhances the bioavailability of other drugs^89,90^, possibly because PP can increase the absorption rate or decrease the elimination rate of combined drugs.

#### Effect of piperine on BDNF and CREB

**Figure 6:**
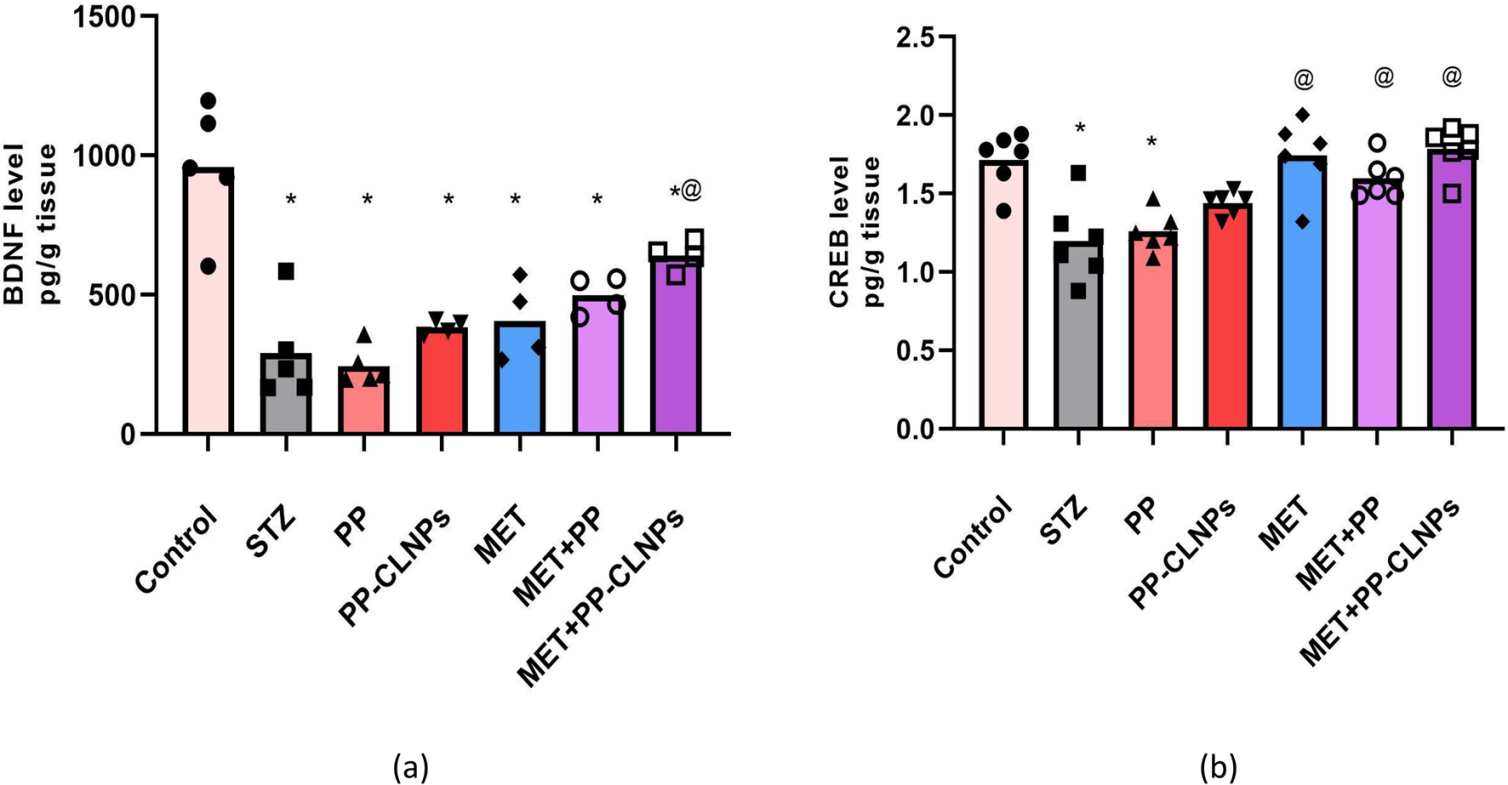
Effect of PP administration on BDNF and CREB levels in serum of diabetic rats. Data represented as mean ± SEM. *Denotes significance from control and # for significance from STZ at p < 0.05. One-way ANOVA followed by Tukey post hoc test was used for analysis.

The present study showed that BDNF expression was inhibited by − 70% in the hippocampus of diabetic rats (p < 0.05) and was not ameliorated by any of the applied treatments except for the combined use of MET and PP-CLNPs, which resulted in 120% increase in BDNF expression (p < 0.05; Fig. 6). STZ caused 30% reduction in CREB in the hippocampus, while treatment with MET or MET in combination with PP or PP-CLNPs restored CREB expression to normal levels (42%, 33%, 48%, p < 0.05), as shown in Fig. 6.

Previous reports have described the anti-inflammatory and antioxidative activities of PP^87,88^. In the present study, the bioformulation of PP-CLNPs with high membrane permeability could be the reason for the superior pharmacological effect of coadministration of MET and PP-chitosan nanoparticles. CLNPs enhance the ability of PPs to interact with multiple molecular targets, including transcription factors, kinases, signalling molecules and inflammatory cytokines^84^. Furthermore, chitosan boosts cellular permeability and promotes intestinal absorption and cellular uptake^85,86^. Moreover, its impact on the absorption rate or rate of elimination of the combined drugs has been proven^89,90^.

The hypoglycaemic effect of metformin is mediated directly by different kinases and promotes the Raf/Ras/Erk/AMPK pathway possibly by inhibiting the mitochondrial respiratory chain and thus increasing the cytoplasmic AMP:ATP ratio, leading to the activation of AMP-activated protein kinase (AMPK)^91^. Indirect effects, including modifying insulin resistance and glycolysis processes, are also involved. However, its cognitive-enhancing effects are not well established. Controversial data are reported from retrospective studies, which hinders the conclusion about its contribution to the control of diabetes-related cognitive impairment or dementia.

In addition to acting as a neurotransmitter modulator and contributing to neuronal plasticity, which is crucial for memory and learning, BDNF is also involved in neuronal survival and growth^92^, while CREB regulates a multitude of brain cellular responses, including proliferation, survival, and differentiation^93^, and is implicated in regulating the transcription of BDNF^94^. Studies have shown that metformin can have a neuroprotective effect on Alzheimer’s disease by increasing BDNF expression through AMPK-mediated CREB phosphorylation. Moreover, metformin-related inhibition of the release of cytochrome *c* from the mitochondria results in increased expression of CREB as a downstream cellular response of AMPK. BDNF and CREB expression are impaired after diabetes induction, as shown in the present study^95,96^. The ameliorating effect of metformin can be explained by the described AMPK mechanism.

been reported that PP induces BDNF in neuro-2α cells^97^. Furthermore, the inhibition of the AMPK pathway was described after PP administration to rats with STZ-induced dementia, which can be linked PP protective action against glutamate-induced apoptosis in rat hippocampus neurons^98,99^. Furthermore, an increase in hippocampal BDNF may be strongly linked to the up-regulation of progenitor cell proliferation of the hippocampal region and the cytoprotective effect of PP^37^. Our data present the first evidence for the cognitive enhancing effect of metformin in which BDNF and CREB signalling are restored and activated to a normal extent after the administration of metformin with PP-CLNPs as an adjuvant.

Induction of oxidative stress is one of the consequential events in diabetes. The accompanying mild cognitive impairment was linked to the presence of high levels of oxidized lipids, DNA, and proteins^100^. In a previous study, PP was shown to exert antioxidant effects, decrease MDA levels, and increase GSH and SOD levels in mice after STZ-induced cognitive impairment^101^. Naturally, PP possess antioxidant properties which impact the morphology and cognitive functioning of neurons positively^102^. It was proven to improve myelin regeneration and memory function in a rat model of demyelination^103^.

#### Behavioural study of the effect of piperine on anxiety and depressive symptoms

##### Effect of piperine on cognitive function

**Figure 7:**
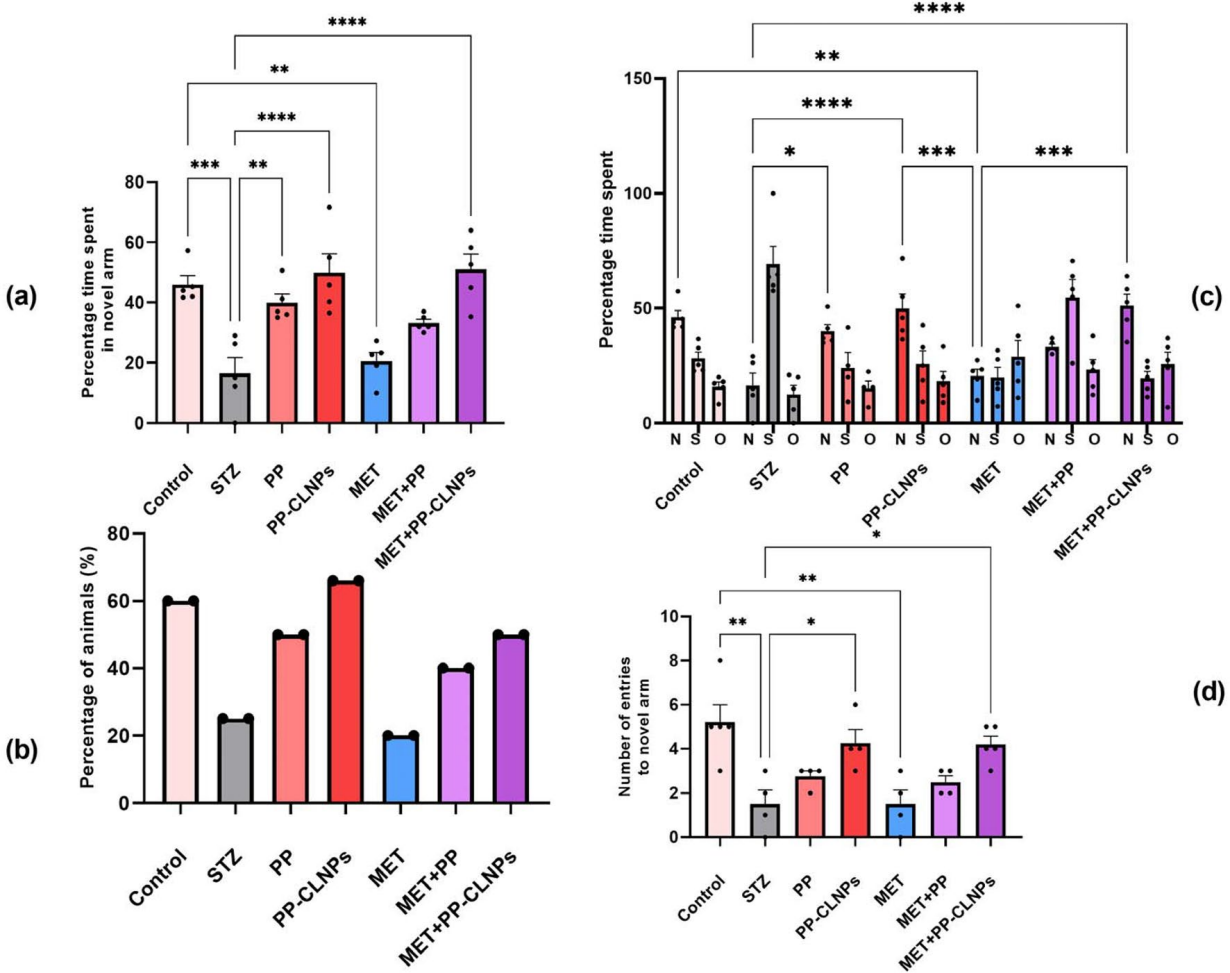
Effect of PP administration on percentage time spent in novel arm and percentage of entries. Data represented as mean ± SEM. *Denotes significance from control and # for significance from STZ at p < 0.05. One-way ANOVA followed by Tukey post hoc test was used for analysis.

Spatial memory is recognized as a marker of cognitive function in rats^104^. Figure 7 reveals that spatial memory was impaired after induction of diabetes by STZ in comparison to nondiabetic animals, as indicated by the percentage of time spent in the novel arm, the number of entries into the novel arm, and the percentage of animals that chose the novel arm as a starting point in the test session. However, the administration of free PP and PP-CLNPs enhanced memory and promoted normal behaviour in diabetic rats. The combination of metformin with PP-CLNPS ameliorated spatial memory disturbance, while the administration of metformin alone had a minimal effect on enhancing cognition. Thus, treatment with PP-CLNPs in combination with metformin could greatly enhance cognitive function. Previous data have shown that the memory-enhancing effect of PP in an AD mouse model and in a cognitive impairment model occurs through antioxidant and cholinergic mechanisms^105,106^. Hippocampus is a crucial brain region for learning and memory particularly consolidation of spatial memory. Piperine was shown to have an impact on acquisition time and retention time as well as escape delay in a water maze test with proven enhanced cognitive impact^107,108^ and was reasoned to be facilitated by the change in serotonin levels^109^.

##### Effect of piperine on latency and immobility time

**Figure 8:**
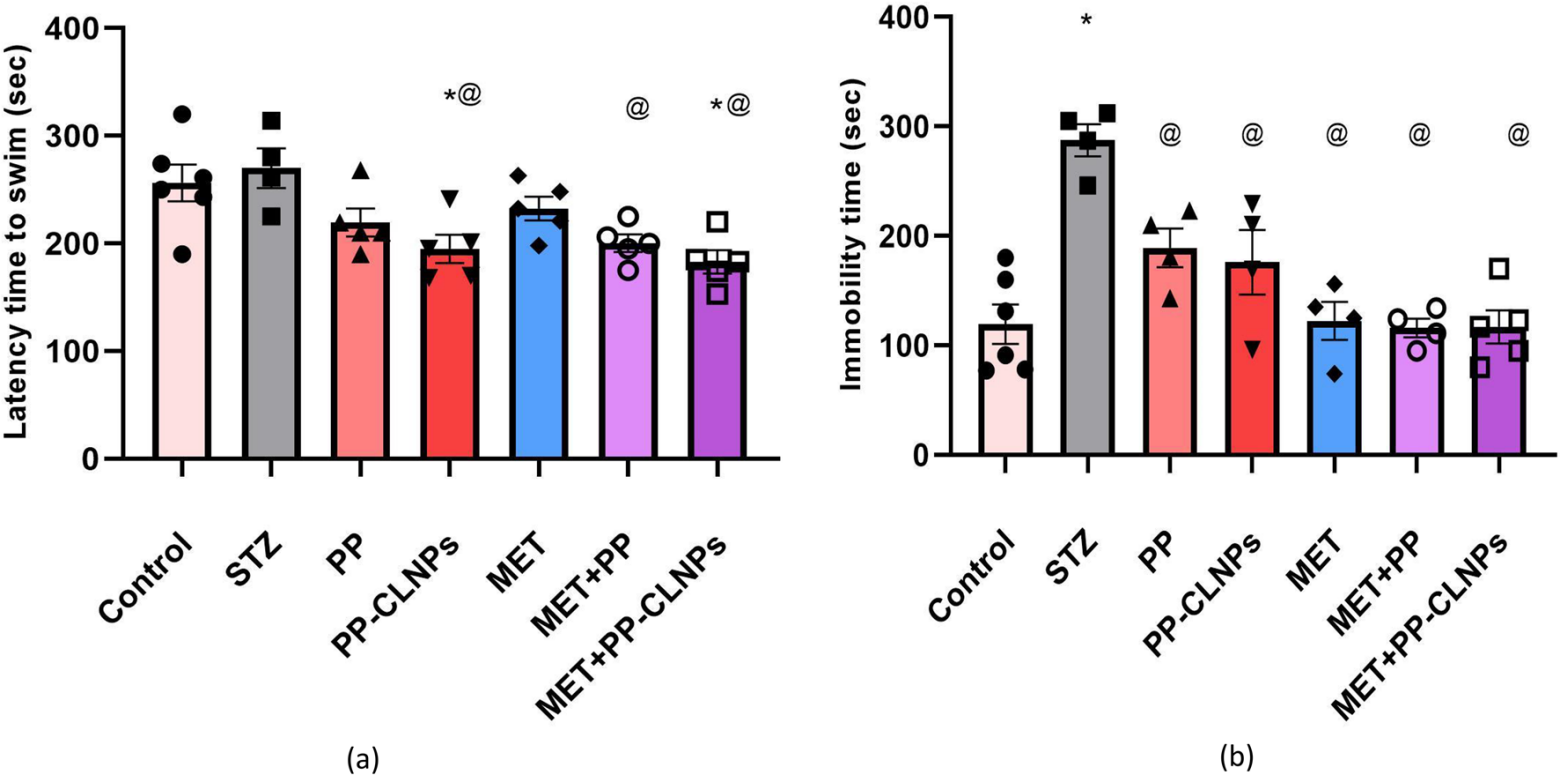
Effect of PP on depressive symptoms in diabetic rats. Data represented as mean ± SEM. *Denotes significance from control and # for significance from STZ at p < 0.05. One-way ANOVA followed by Tukey post hoc test was used for analysis.

According to Roy and Lloyd^110^, type 2 diabetic individuals may experience depression at rates up to twice as high as the global average. Therefore, the creation of innovative antidiabetic regimens that also have antidepressive effects would offer a fresh perspective on drug discovery. It was previously mentioned that STZ administration can result in an increase in the latency to swim and immobility time in the forced swim test, signifying a pro-depressant effect^111^. Figure 8 reveals that treatment with free PP or PP-CLNPs significantly decreased the latency and immobility time compared to those of STZ-treated diabetic rats and control nondiabetic rats. MET treatment could normalize the immobility time. Cotreatment with MET and PP-CLNPs enhanced the ability of MET to normalize immobility time.

##### Effects of piperine on horizontal and vertical locomotor activity

**Figure 9:**
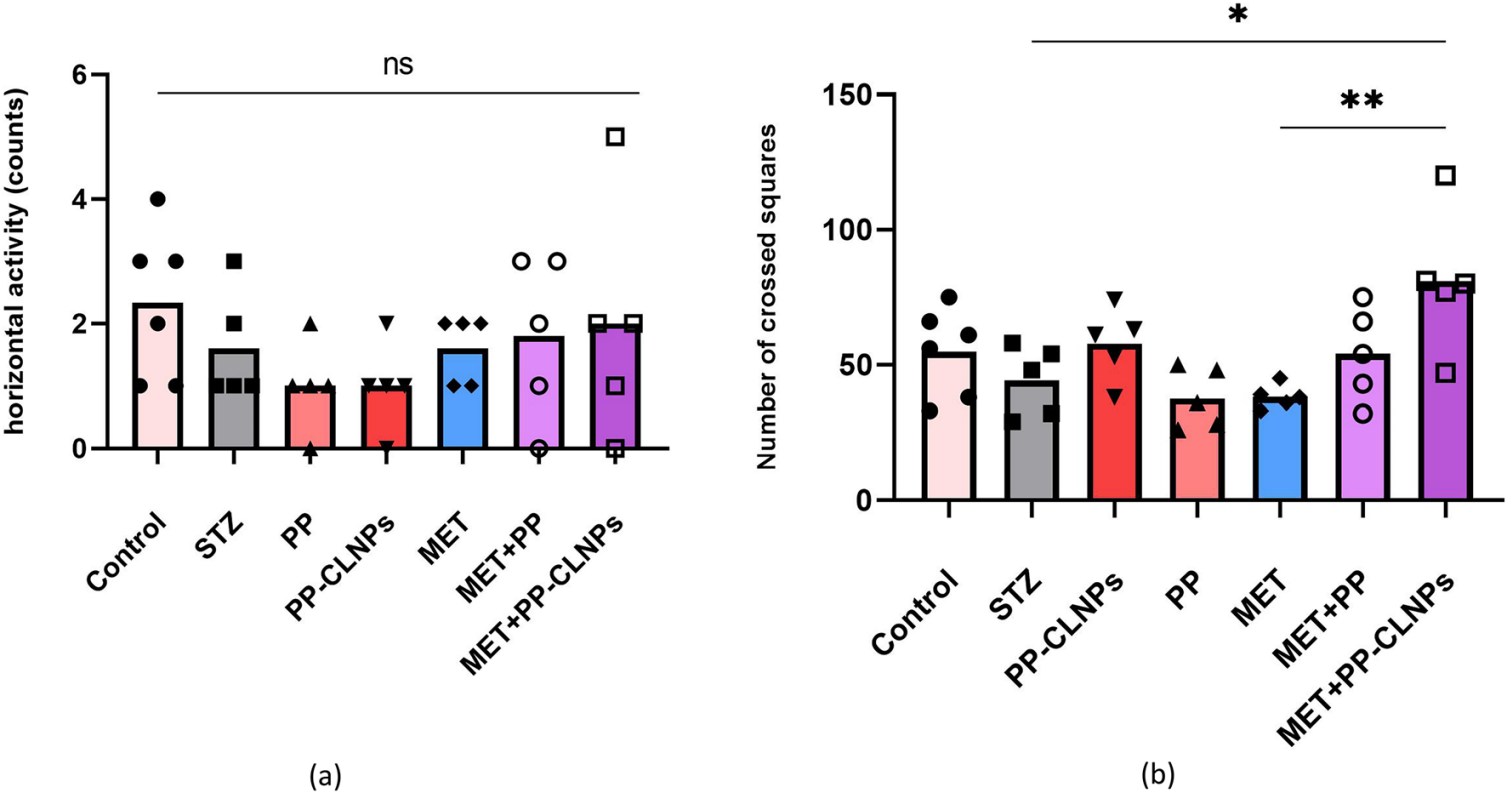
Effect of PP administration on (**a**) number of squares crossed (horizontal locomotor activity and (**b**) number of rears (vertical locomotor activity). Data represented as mean ± SEM. *Denotes significance from control and # for significance from STZ at p < 0.05. One-way ANOVA followed by Tukey post hoc test was used for analysis.

Diabetes leads to multiple modifications in the morphology and function of the central nervous system, leading to a decrease in mobility and locomotor activity^112^. Figure 9 shows the effects of the different treatments on locomotor activity. Generally, locomotor activity was the same among the treatment groups. The combination of MET and PP-CLNPs strongly promoted locomotor activity, which was significantly different from that in the diabetic group. This enhanced effect may be because PP can offer neuroprotective support in diabetic rats^113,114^.

##### Effect of piperine on anxiety in the elevated plus maze

**Figure 10:**
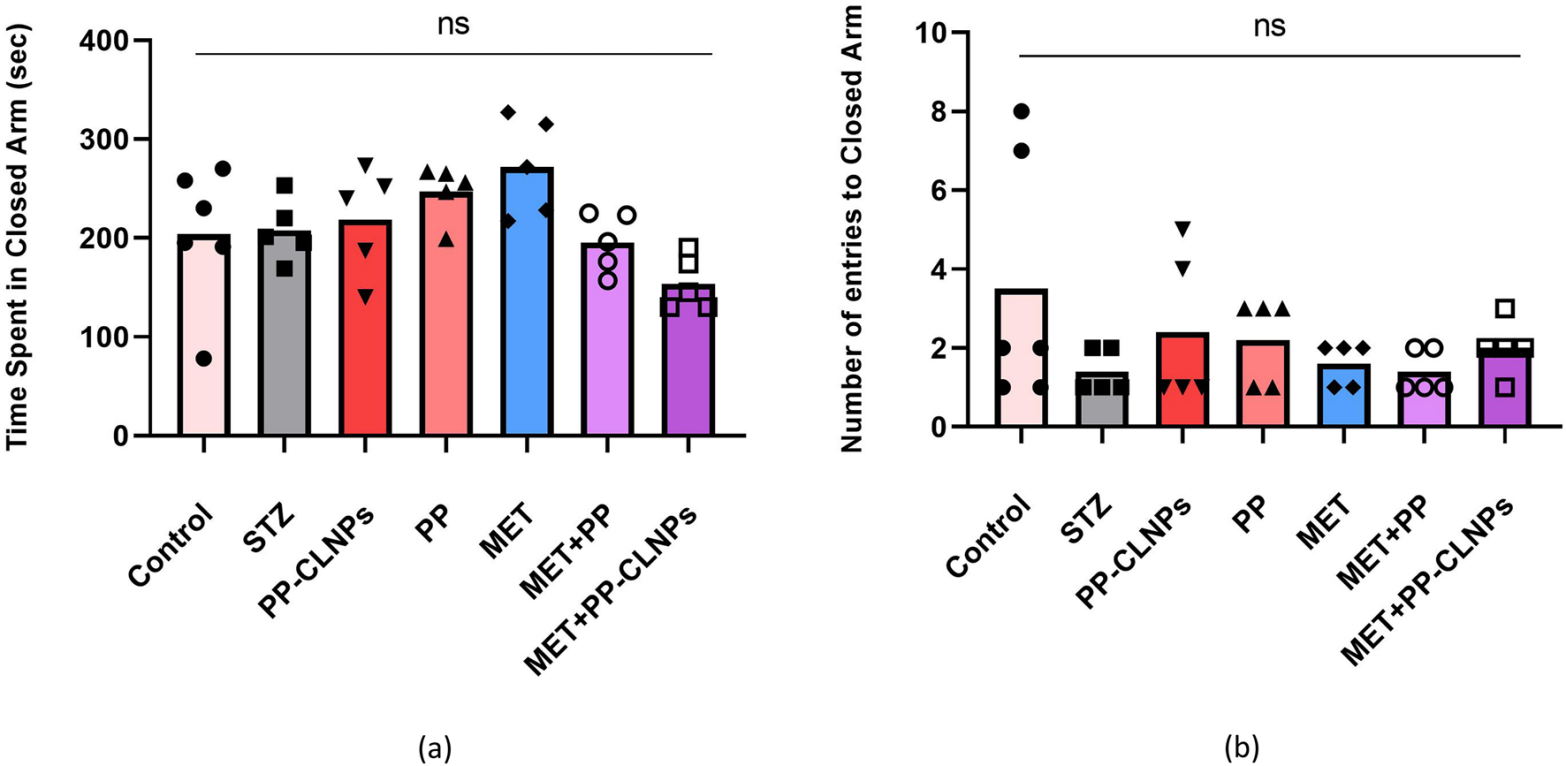
Effect of PP administration on anxiety behavior in elevated plus maze. (**a**) Time spent in closed arm, (**b**) Number of entries to closed arm. Data represented as mean ± SEM. *Denotes significance from control and # for significance from STZ at p < 0.05. One-way ANOVA followed by Tukey post hoc test was used for analysis.

It has been reported that diabetes can induce behavioural changes, such as anxiety and depression, in patients, suggesting the induction of a depressive state^115^. Figure 10 shows that, compared with those in the STZ group, the diabetic rats in the elevated plus maze group displayed increased anxiety and were more likely to spend more time in the open arms. Administration of PP with MET resulted in a reasonable increase in the number of entries and time spent in the open arms. Treatment with PP improved social behaviour in MET-treated rats. The protective effects of PP on the amygdala, its antianxiety effect, and its capacity to prevent glutamate release in nerve terminals may all contribute to its ability to ameliorate behavioural changes^116^.

## Conclusion and future prospectives

In the present study, PP was successfully loaded in CLNPs prepared using chitosan, stearic acid, Tween 80 and TPP at different concentrations. The prepared nanoparticles were all in the nanosize range and exhibited high ability to encapsulate PP and negative ZP values. The formulations F2, F5, and F8 exhibited high EE%, suitable VS and ZP values and accordingly they were selected for further investigation. PP release from the selected formulations was biphasic in nature that followed a diffusion-controlled release kinetic model. In vivo studies revealed that treatment with the optimized PP-CLNPs formulation (F2) exerted a cognitive enhancing effect and ameliorated the oxidative stress associated with diabetes. Piperine acted as an effective bio-enhancer and thus increased the potency of metformin in protecting brain tissue from diabetes-induced neuroinflammation and memory deterioration. Its bio-enhancer activity can be possibly through augmenting the AMPK pathway through its downstream neuroplasticity targeting CREB and BDNF. The obtained results suggested that PP-CLNPs can be used as an adjuvant therapy in the management of high-risk diabetic cognitive impairment conditions and we suggest that PP-CLNPs can be further investigated as an adjuvant therapy with other antidiabetic agents to protect and control high-risk diabetic cognitive impairment conditions and/or other diabetic chronic complications.
